# Supplementary material for: Mechanisms governing the pioneering and redistribution capabilities of the non-classical pioneer PU.1
Source: Nat Commun. 2020 Jan 21;11:402. doi: 10.1038/s41467-019-13960-2 (PMC6972792; doi:10.1038/s41467-019-13960-2)
Supplement: Supplementary file 7 — Source data [file 41467_2019_13960_MOESM7_ESM.zip › Source_Data/Figure5/Figure5A_MotifScanOutput/homerResults/motif14.similar.html]

motif14

## Information for motif14

G
C
T
A
G
C
T
A
A
G
C
T
T
G
A
C
C
G
T
A
T
G
A
C
C
G
T
A
A
C
T
G
G
T
A
C
C
G
A
T
A
C
T
G
G
A
C
T
A
C
G
T
G
C
T
A
  
Reverse Opposite:  

C
G
A
T
T
G
C
A
C
T
G
A
T
G
A
C
C
G
T
A
C
A
T
G
G
T
A
C
A
C
G
T
A
C
T
G
C
G
A
T
A
C
T
G
T
C
G
A
C
G
A
T
C
G
A
T
  

|  |  |
| --- | --- |
| p-value: | 1e-53 |
| log p-value: | -1.224e+02 |
| Information Content per bp: | 1.581 |
| Number of Target Sequences with motif | 722.0 |
| Percentage of Target Sequences with motif | 23.94% |
| Number of Background Sequences with motif | 5996.6 |
| Percentage of Background Sequences with motif | 13.15% |
| Average Position of motif in Targets | 247.4 +/- 187.0bp |
| Average Position of motif in Background | 210.6 +/- 142.4bp |
| Strand Bias (log2 ratio + to - strand density) | -0.0 |
| Multiplicity (# of sites on avg that occur together) | 1.15 |
| Motif File: | file (matrix) reverse opposite |

### Similar de novo motifs found

|  |  |  |  |  |  |  |  |
| --- | --- | --- | --- | --- | --- | --- | --- |
| Rank | Match Score | Redundant Motif | P-value | log P-value | % of Targets | % of Background | Motif file |
| 1 | 0.882 | C T G A T A G C C G T A C A T G G T A C G C A T A C T G G A C T T G A C | 1e-42 | -98.939899 | 30.07% | 19.17% | motif file (matrix) |
| 2 | 0.784 | G A C T A G C T C G A T C A G T T G A C C G T A T G A C G C T A A C T G G T A C G C T A T C A G | 1e-38 | -87.504716 | 56.40% | 44.22% | motif file (matrix) |
